# Towards a Prophylactic Vaccine for the Prevention of HCMV Infection

**DOI:** 10.3390/vaccines9090968

**Published:** 2021-08-29

**Authors:** Gaëtan Ligat, Sophie Alain, Sébastien Hantz

**Affiliations:** 1 Institut de Recherche sur les Maladies Virales et Hépatiques, Université de Strasbourg, 67000 Strasbourg, France; 2 INSERM, CHU Limoges, University of Limoges, RESINFIT, U1092, 87000 Limoges, France; sophie.alain@unilim.fr (S.A.); sebastien.hantz@unilim.fr (S.H.); 3 CHU Limoges, Laboratoire de Bactériologie-Virologie-Hygiène, National Reference Center for Herpesviruses (NRCHV), 87000 Limoges, France

Human cytomegalovirus (HCMV) or human herpesvirus 5, is a ubiquitous human herpesvirus, which can cause severe disease in immunocompromised patients (AIDS patients and solid organ transplant or hematopoietic stem cell transplant recipients). HCMV is also the most common infectious cause of congenital malformations, with developmental delay, sensorineural hearing loss and fetal death in 10–15% of cases [1]. Three major molecules, ganciclovir (GCV), cidofovir (CDV) and foscarnet (FOS), all targeting the HCMV polymerase pUL54, are routinely used for the prevention and treatment of HCMV infection in the transplant setting. 

In these patients, the emergence of HCMV drug resistance, favored by long-term exposure to antiviral drugs, low doses, and prolonged immunosuppression, is a growing therapeutic challenge that is added to the toxicity of the molecules. Resistance mutations occur in the UL97 kinase (leading to GCV resistance) or in the UL54 polymerase, leading to various levels of cross-resistance (GCV, FOS, CDV). As they have hematological and renal toxicity, these drugs are not recommended for administration in pregnant women and their use could be limited in transplant settings [2].

Recent attempts to develop new anti-HCMV compounds with lower toxicity, such as maribavir and letermovir, have focused mainly on novel targets respectively, the viral kinase pUL97 and the viral terminase complex involved in viral DNA cleavage/packaging [3,4,5]. Letermovir received European and U.S. approval for prophylactic use in hematopoietic stem cell transplants in 2017. However, the emergence of resistance mutations against both drugs have been already described [6].

In the last decade, the main challenge in the field of medical virology has been the development of prophylactic vaccines for the prevention of HCMV infection. A vaccination against HCMV could protect immunocompromised patients and prevent birth defects caused by congenital HCMV infections. Thus, HCMV prophylactic vaccine development is a major public health priority. One of the main obstacles to the development of an efficient vaccine is the lack of protection provided by immune memory cells against HCMV re-activation and re-infection.

To date, candidate vaccines for entry into clinical evaluation are based on modified and attenuated viral strains, protein combinations based on viral glycoproteins including glycoprotein B (gB) [7], vector-based vaccination approaches allowing for the expression of antigens such as glycoprotein B and/or phosphoprotein pp65. A more recent vaccine strategy relies on immunization from synthetic peptides mimicking the major epitopes of the immunogenic viral proteins. The first trials date back to the 1970s. The approaches were then concentrated on the development of attenuated virus-based vaccines produced from the HCMV reference strain Towne and AD169 [8,9]. A phase 2 study of V160 vaccine is underway (attenuated AD169-based vaccine developed by Merck) [10]. Another approach has been the development of a chimeric vaccine exhibiting the characteristics of the attenuated Toledo and Towne strains. The chimeric vaccine candidates were well tolerated and did not cause systemic infection [11,12]. Other studies have used a gB vaccine based on recombinant gB proteins which in turn has the potential to decrease the incident cases of maternal and congenital HCMV infection [13]. However, the efficacity of such strategies is limited. The quick development of mRNA-based vaccines to protect against novel severe acute respiratory syndrome coronavirus 2 (SARS-CoV-2) during the COVID-19 pandemic led to the development of mRNA-based vaccines both for oncology and infectious diseases, including HCMV infections. A HCMV mRNA vaccine was first developed by Novartis (now GSK) and contained gB and a pp65-IE1 fusion construct [14,15]. Other mRNA-based vaccines, developed by Moderna, Inc encoded HCMV gB and pentameric complex (PC) or the major HCMV T-cell antigen pp65. Such vaccines elicit potent humoral and cell-mediated immunity that can be used in a heterologous prime/ boost vaccination regimen with PC and gB to broaden T cell responses [16]. More recently, phase 2 clinical trials confirmed preliminary results with the vaccine combining six mRNAs in a single vial, which encode for two antigens located on the surface of HCMV: five mRNAs encoding the subunits that form the membrane-bound pentamer complex and one mRNA encoding the full-length membrane-bound glycoprotein B (gB). This vaccine continues its clinical evaluation in phase 3 clinical trials.

Despite many advances in HCMV prophylactic vaccine development, a better understanding of the maternal-placental-fetal triad is needed to identify potential targets. Indeed, the determination of the immunological and virological parameters which correlate with protection from transmission at the maternal-fetal interface is essential for the optimization of available or future vaccine candidates. Finally, population studies of HCMV transmission to childbearing age women and of circulating strains will help to better define target populations for these new vaccines.

## Data Availability

Not applicable.
